# Identification of osteoporosis ferroptosis-related markers and potential therapeutic compounds based on bioinformatics methods and molecular docking technology

**DOI:** 10.1186/s12920-024-01872-0

**Published:** 2024-04-22

**Authors:** Shi-Wei Long, Shi-Hong Li, Jian Li, Yang He, Bo Tan, Hao-Han Jing, Wei Zheng, Juan Wu

**Affiliations:** 1 Department of Orthopedic Oncology, Shanghai Sixth People’s Hospital Affilicated to Shanghai Jiao Tong University School of Medicine, Shanghai, China; 2 Southwest Jiao Tong University School of Medicine, Chengdu, China; 3 General Hospital of Western Theater Command, Chengdu, China

**Keywords:** Ferroptosis, Osteoporosis, GEO, Random forest, Molecular docking

## Abstract

**Research background and purpose:**

Osteoporosis (OP) is one of the most common bone diseases worldwide, characterized by low bone mineral density and susceptibility to pathological fractures, especially in postmenopausal women and elderly men. Ferroptosis is one of the newly discovered forms of cell death regulated by genes in recent years. Many studies have shown that ferroptosis is closely related to many diseases. However, there are few studies on ferroptosis in osteoporosis, and the mechanism of ferroptosis in osteoporosis is still unclear. This study aims to identify biomarkers related to osteoporosis ferroptosis from the GEO (Gene Expression Omnibus) database through bioinformatics technology, and to mine potential therapeutic small molecule compounds through molecular docking technology, trying to provide a basis for the diagnosis and treatment of osteoporosis in the future.

**Materials and methods:**

We downloaded the ferroptosis-related gene set from the FerrDb database (http://www.zhounan.org/ferrdb/index.html), downloaded the data sets GSE56815 and GSE7429 from the GEO database, and used the R software “limma” package to screen differentially expressed genes (DEGs) from GSE56815, and intersected with the ferroptosis gene set to obtain ferroptosis-related DEGs. Gene Ontology (GO) and Kyoto Encyclopedia of Genes and Genomes (KEGG) analysis were performed by the R software “clusterProfiler” package. The random forest model was further screened to obtain essential ferroptosis genes. R software “corrplot” package was used for correlation analysis of essential ferroptosis genes, and the Wilcox test was used for significance analysis. The lncRNA-miRNA-mRNA-TF regulatory network was constructed using Cytoscape software. The least absolute shrinkage and selection operator (LASSO) was used to construct a disease diagnosis model, and a Receiver operating characteristic (ROC) curve was drawn to evaluate the diagnostic performance, and then GSE7429 was used to verify the reliability of the diagnosis model. Molecular docking technology was used to screen potential small molecule compounds from the Drugbank database. Finally, a rat osteoporosis model was constructed, and peripheral blood mononuclear cells were extracted for qRT-PCR detection to verify the mRNA expression levels of crucial ferroptosis genes.

**Result:**

Six DEGs related to ferroptosis were initially screened out. GO function and KEGG pathway enrichment analysis showed that ferroptosis-related DEGs were mainly enriched in signaling pathways such as maintenance of iron ion homeostasis, copper ion binding function, and ferroptosis. The random forest model identified five key ferroptosis genes, including *CP*, *FLT3*, *HAMP*, *HMOX1*, and *SLC2A3*. Gene correlation analysis found a relatively low correlation between these five key ferroptosis genes. The lncRNA-miRNA-mRNA-TF regulatory network shows that *BAZ1B* and *STAT3* may also be potential molecules. The ROC curve of the disease diagnosis model shows that the model has a good diagnostic performance. Molecular docking technology screened out three small molecule compounds, including NADH, Midostaurin, and Nintedanib small molecule compounds. qRT-PCR detection confirmed the differential expression of *CP*, *FLT3*, *HAMP*, *HMOX1* and *SLC2A3* between OP and normal control group.

**Conclusion:**

This study identified five key ferroptosis genes (*CP*, *FLT3*, *HAMP*, *HMOX1*, and *SLC2A3*), they were most likely related to OP ferroptosis. In addition, we found that the small molecule compounds of NADH, Midostaurin, and Nintedanib had good docking scores with these five key ferroptosis genes. These findings may provide new clues for the early diagnosis and treatment of osteoporosis in the future.

**Supplementary Information:**

The online version contains supplementary material available at 10.1186/s12920-024-01872-0.

## Introduction

Due to the aging population and increasing life expectancy, osteoporosis is a serious public health problem worldwide [1]. Osteoporosis (OP) is a systemic metabolic bone disease characterized by decreased bone mineral density, altered bone microarchitecture, decreased bone mass, and susceptibility to fragility fractures [2]. Iron synthesizes various important proteases and is the essential element of body life activities [3, 4]. Ferroptosis is a newly discovered way of programmed cell death, which is different from other types of programmed cell death (such as apoptosis, pyroptosis, and autophagy), which is characterized by excessive accumulation of iron-dependent lipid peroxides leading to cell death [5]. Ferroptosis has three essential features: the presence of redox-active iron, dysfunction of lipid peroxide clearance, and oxidation of phospholipids containing unsaturated fatty acids [5–7]. Many studies have shown that it is associated with various diseases [8–12], such as tumors, osteoporosis, immune response, viral infection, Parkinson’s disease, atherosclerosis, ischemia-reperfusion injury [13–15], ferroptosis is expected to become a new research direction for disease treatment. It has been proven that some drugs for the treatment of diabetes affect bone metabolism and increase the risk of fractures [16, 17]. It has been reported that targeting ferroptosis can significantly reverse diabetic osteoporosis (DOP)-associated bone loss and bone cell death, ultimately improving trabecular bone deterioration. In addition, glucocorticoid-induced osteoporosis is also closely related to ferroptosis [18]. There is increasing research on the relationship between iron and osteoporosis, and it is now found that disorders of iron metabolism, including iron deficiency and iron overload, can lead to osteoporosis [19–23]. However, the specific mechanism of ferroptosis in osteoporosis remains unclear. For the treatment of osteoporosis, drug therapy is currently the mainstay [24], but the discovery and development of anti-osteoporosis drugs are often time-consuming, high-risk, and expensive. In recent years, the increasing application of combinatorial chemistry and high-throughput screening techniques has facilitated drug discovery [25]. Molecular docking technology is widely used in drug discovery and plays a vital role in predicting the relationship between molecules and biological targets [26]. Molecular docking technology helps to expand the different indications of drugs and discover multiple uses of drugs. In this study, we will use the above techniques to explore biomarkers related to ferroptosis in osteoporosis, which provides a new direction for the early diagnosis and treatment of osteoporosis.

In this study, bioinformatics technology was used to mine OP-related gene expression profiles from the GEO database, and R software was used to screen out ferroptosis-related differentially expressed genes (DEGs), GO (Gene Ontology) and KEGG (Kyoto Encyclopedia of Genes and Genomes) enrichment analyses were performed on them. Subsequently, the key ferroptosis genes were further determined by constructing a random forest model, and the expression correlation analysis of the key ferroptosis genes was carried out. We made a lncRNA (long non-coding RNA)-miRNA (micro-RNAs)-mRNA (Messenger RNA)-TF (transcription factors) regulatory network to understand the underlying molecular mechanisms further. At the same time, we used key ferroptosis genes in LASSO (Least absolute shrinkage and selection operator) regression to construct a disease diagnostic model and external datasets to validate the diagnostic model. In addition, to mine potential therapeutic compounds for osteoporosis, we used molecular docking technology to screen out small molecule compounds with good docking scores with key ferroptosis genes from the DrugBank database. Finally, mononuclear cells in the peripheral blood of osteoporosis model mice were extracted, and qRT-PCR (Quantitative Real-time PCR) experiments were performed to detect the mRNA expression levels of key ferroptosis genes in the disease, and the expression results of chip data analysis were verified. This study is expected to provide new biomarkers for the early diagnosis of osteoporosis in the future and provide a theoretical basis for exploring the occurrence and development of osteoporosis and the discovery of new drugs. Currently, there are few studies related to ferroptosis OP, and even fewer studies on improving OP through the ferroptosis pathway. Therefore, the compounds discovered in our study are very promising in the treatment of osteoporosis.

## Method

### Data collection and organization

This study downloaded the GSE56815 dataset from the GEO database as a training set and the GSE7429 dataset from the website as a validation set for model diagnostic efficacy. Among them, GSE56815 contains blood samples of 40 women with high BMD (bone mineral density) and 40 samples of women with low BMD, while GSE7429 contains blood samples of 10 women with high BMD and ten blood samples of women with low BMD. The downloaded data sets GSE56815 and GSE7429 are expression profiles after data normalization. The probe IDs are corresponding to the genes, and the empty probes are removed. If multiple probes correspond to the same gene, the median is selected as the expression level of the gene. First, we downloaded ferroptosis-related gene sets from the FerrDb database (http://www.zhounan.org/ferrdb/index.html). These genes include drivers, inhibitors, and markers. In addition, three ferroptosis gene sets were collected from published studies, including PMID: 32,760,210, PMID: 33,330,074, and PMID: 33,867,820. Finally, these ferroptosis gene sets were combined to obtain a final total of 376 ferroptosis genes, which were used for subsequent analysis.

### Identification of differentially expressed genes related to ferroptosis

We used the “limma” package in R software to perform differential genetic analysis on the processed data, and the screening threshold was set to an adjusted *p*-value < 0.05 and |log_2_FC| >0. The DEGs and ferroptosis-related gene sets obtained through screening were imported into the online Venny 2.1 (https://bioinfogp.cnb.csic.es/tools/venny/) to obtain a Venn plot of ferroptosis-related differentially expressed genes.

### GO and KEGG enrichment analysis

We used the “clusterProfiler” (version 4.6.2) and “org.Hs.eg.db” packages of R software to conduct GO function and KEGG pathway enrichment analysis on ferroptosis-related differentially expressed genes. The screening standard is *p*.adjust < 0.05, and the enrichment analysis results are displayed visually.

### Random forest analysis to screen key ferroptosis genes

Random forest (RF) is one of the most widely used machine learning methods and is often used to screen differential genes. We input the 6 DEGs related to ferroptosis into the random forest classifier, used the R software “randomForest” package (version 4.7.1.1) to build the random forest model, and selected the appropriate mtry value and ntree value as the two parameters to build the random forest. The key parameter model was used to obtain the Mean Decrease Accuracy value and the Mean Decrease Gini value, and the essential genes for ferroptosis were determined based on these two values.

### Correlation analysis among key ferroptosis genes

The “corrplot” package (version 0.92) of R software was used to perform correlation analysis on key ferroptosis genes, and the correlation coefficient between genes was calculated. This study also used the Wilcox test to analyze the significance of key ferroptosis genes. When *p* < 0.05, it was considered to be significantly correlated, and crosses represented those that did not pass the significance test in the heat map.

### Construction of the lncRNA-miRNA-mRNA-TF regulatory network

This study collected lncRNAs, miRNAs, and TFs that interact with five key ferroptosis genes from the Starbase (https://starbase.sysu.edu.cn/), TRRUST (https://www.grnpedia.org/trrust/), and mirDIP (http://ophid.utoronto.ca/mirDIP/), respectively. We used Cytoscape software (version 3.8.1) to visualize the sorting data on the lncRNA-miRNA-mRNA-TF regulatory network.

### Construction of the disease diagnosis model and drawing ROC curve

LASSO is one of the commonly used machine learning algorithms, which can be used to build clinical prediction models. In this study, key ferroptosis genes were used to construct a diagnostic model (analysis method: binomial, cross-validation method: auc, cross-validation multiple: 10), The coef value (Coefficients) of each gene was returned based on the expression of these five genes in the input training set and the diagnostic outcome. The coef value was the basic coefficient in front of each gene in the model diagnostic formula. The score of this model was the sum of the expression of each gene multiplied by its coefficient, which can be used for disease diagnosis. The ROC (receiver operating characteristic) curve was drawn using the R software “ROCR” package (version 1.0.11) and the area under the curve (AUC) value was calculated to evaluate the diagnostic performance of the model. Finally, the reliability of the diagnostic model was verified by the external validation set GSE7429.

### Molecular docking screening of small molecule compounds

First, the protein structure corresponding to the gene came from the PDB database (https://www.rcsb.org/), and the spatial structure of small molecule compounds came from the DrugBank database (https://go.drugbank.com/). We used AutoDock Vina software to simulate the binding affinity of protein-ligands (genes and small molecule compounds). The downloaded protein was deleted from water molecules, polar hydrogen and Coleman charges are added, and the model with the lowest binding energy (Affinity ≤ -7 kcal/mol) was selected for subsequent analysis. Finally, Pymol software was used to optimize and visualize the molecular docking results.

### Construction of osteoporosis model

The Ethics Committee of the Western Theater Command General Hospital fully approved this study. All methods were carried out in accordance with ARRIVE guidelines (https://arriveguidelines.org) for the reporting of animal experiments and the guidelines of the Western Theater Command General Hospital on the ethical use of animals. Ten female SD rats aged 6–8 weeks (purchased from Chengdu Dashuo Experimental Animal Co., Ltd.) were randomly divided into a sham operation (Sham) group and an ovariectomy (OVX) group, with five rats in each group. Ethical approval to conduct the study was obtained from The Institutional Animal Care and Use Committee at General hospital of Western Theater. Two groups of rats were operated on to construct an animal model, and its steps are as follows. After anesthesia, the OVX group removed both ovaries and part of the fallopian tubes, while the Sham group retained the ovaries and only removed the same-sized fat around the ovaries. The postoperative rats were fed in groups, and they ate normally during the feeding period, and the feeding cycle was 12 weeks.

### Micro-CT detection

After the Sham and OVX group rats were sacrificed, the femurs were taken out and the attached muscles and other soft tissues were removed. The clean femurs were soaked and fixed in 4% paraformaldehyde solution for 24 h and then analyzed by Micro-CT. Use the 3D reconstruction software NRecon (software version V1.7.4.2, Bruker, Germany) to reconstruct the selected area of the original image. CT Analyzer (software version 1.20.3.0, Bruker, Germany) was used to analyze the ROI in the 5 mm region below the proximal femoral epiphysis of rats. A unified parameter was set, and the bone microstructural parameters of the bone tissue in the target area are calculated by the software.

### qRT-PCR detection

First, whole blood was collected from rats in the Sham group and OVX group respectively, monocytes were extracted according to the instructions of rat peripheral blood mononuclear cell isolation kit (Suolaibao, Beijing, China), total RNA was extracted with Trizol reagent, and reverse cDNA was synthesized using a recording kit (Takara, Dalian, China). GAPDH was used as an internal reference gene to obtain uniformly expressed mRNA. All samples were repeated three times, and the final results were calculated using the 2^−ΔΔCq^ method. The primer sequences used in the experiments are shown in Table 1.

**Table 1:** PCR primer sequence

Gene	Primer sequence (5’-3’)
SLC2A3-R	GATGTCACAGGAGAAGCAGGTCAC
SLC2A3-F	TGGAGGACAACGGAGATGAGAAGG
HMOX1-R	GGGTCAGGTGTCCAGGGAAGG
HMOX1-F	TGGGTTCTGCTTGTTTCGCTCTATC
HAMP-R	AAGGCAAGATGGCACTAAGCACTC
HAMP-F	GCCGTAGTCTGTCTCGTCTGTTG
FLT3-R	AAGAGGCTGGAAGAAGAGGAGGAAG
FLT3-F	GCTGCCAGGTCTCTGTGAACAC
CP-R	TGCGTGCCAATGAGCCAAGTC
CP-F	CAACGGTCCTATGAGTCCTGATGC
GAPDH-R	GCAGAATTCCTGGCCAAGGTCATCCATGAA
GAPDH-F	GCAGGTACCGGGGCCATCCACAGTCTTCTG

### Statistical analysis methods

For the experimental data, the mean ± standard deviation was used to represent. Differences between the two independent groups were assessed using Student’s t-test (unpaired). GraphPad Prism software (version 9.4.0) was used for statistical analysis and drawing, and when *P* < 0.05, the difference was considered statistically significant.

## Results and analysis

### Identification of differentially expressed genes related to ferroptosis

**Fig. 1:**
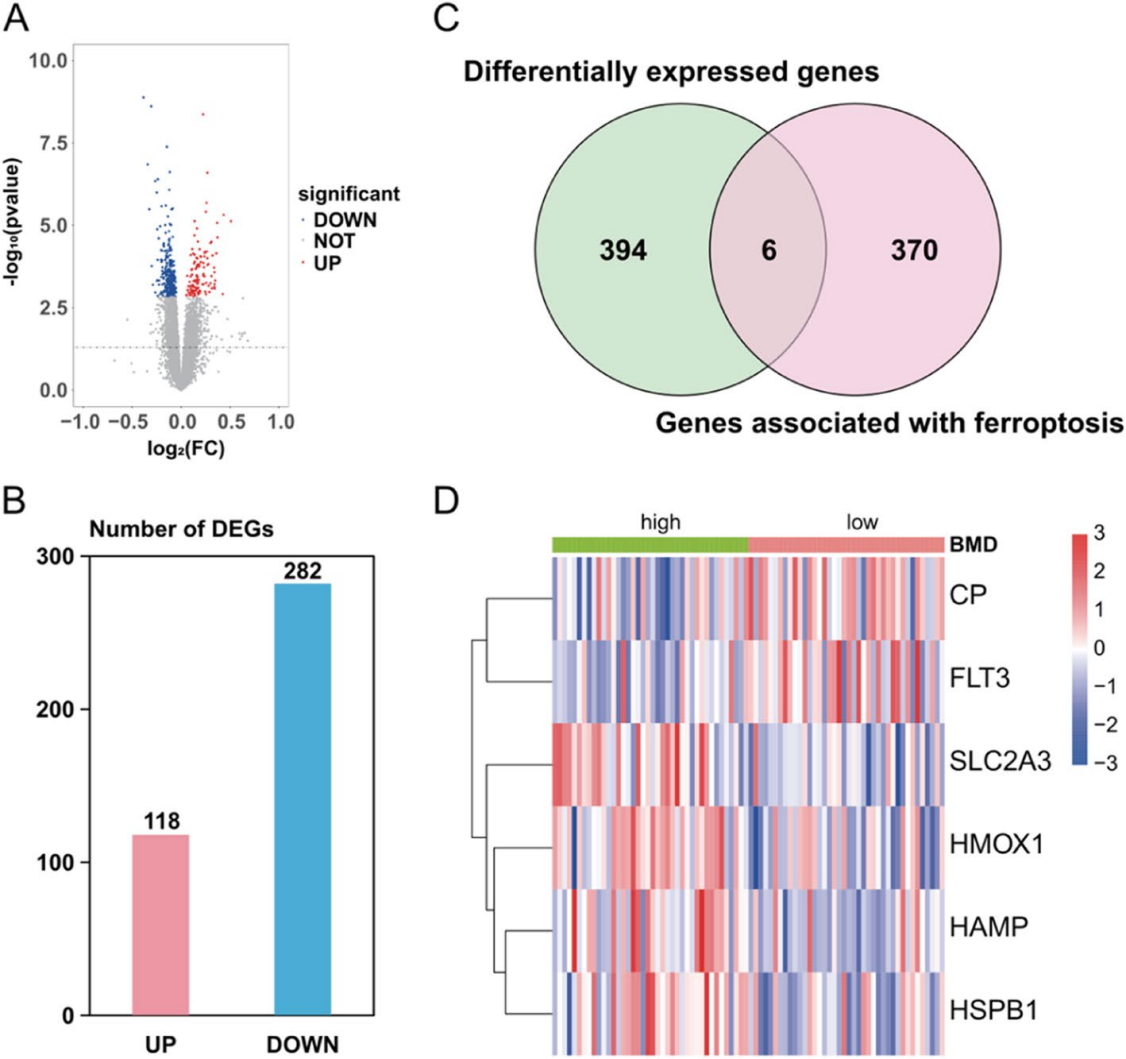
GSE56815 dataset gene differential expression analysis. Note: **A** Volcano map of DEGs in the GSE56815 dataset, red dots represent up-regulated genes, blue dots represent down-regulated genes, and gray dots represent insignificant genes. **B** Statistical histogram of the number of differentially expressed genes in the GSE56815 dataset. **C** Venn diagram of DEGs and ferroptosis-related genes. **D** Six ferroptosis-related DEGs clustered heat map in the GSE56815 dataset, the red part indicates the gene’s high expression, the blue part indicates low gene expression, and the white part indicates no significant change

Through differential expression analysis, a total of 400 DEGs were screened from the dataset GSE56815, including 118 up-regulated genes and 282 down-regulated genes (Fig. 1A and B). The obtained 376 ferroptosis-related genes were intersected with 400 DGEs to obtain six ferroptosis-related DEGs, as shown in the Venn diagram and heat map of ferroptosis-related DEGs (Fig. 1C and D).

### GO and KEGG analysis

**Fig. 2:**
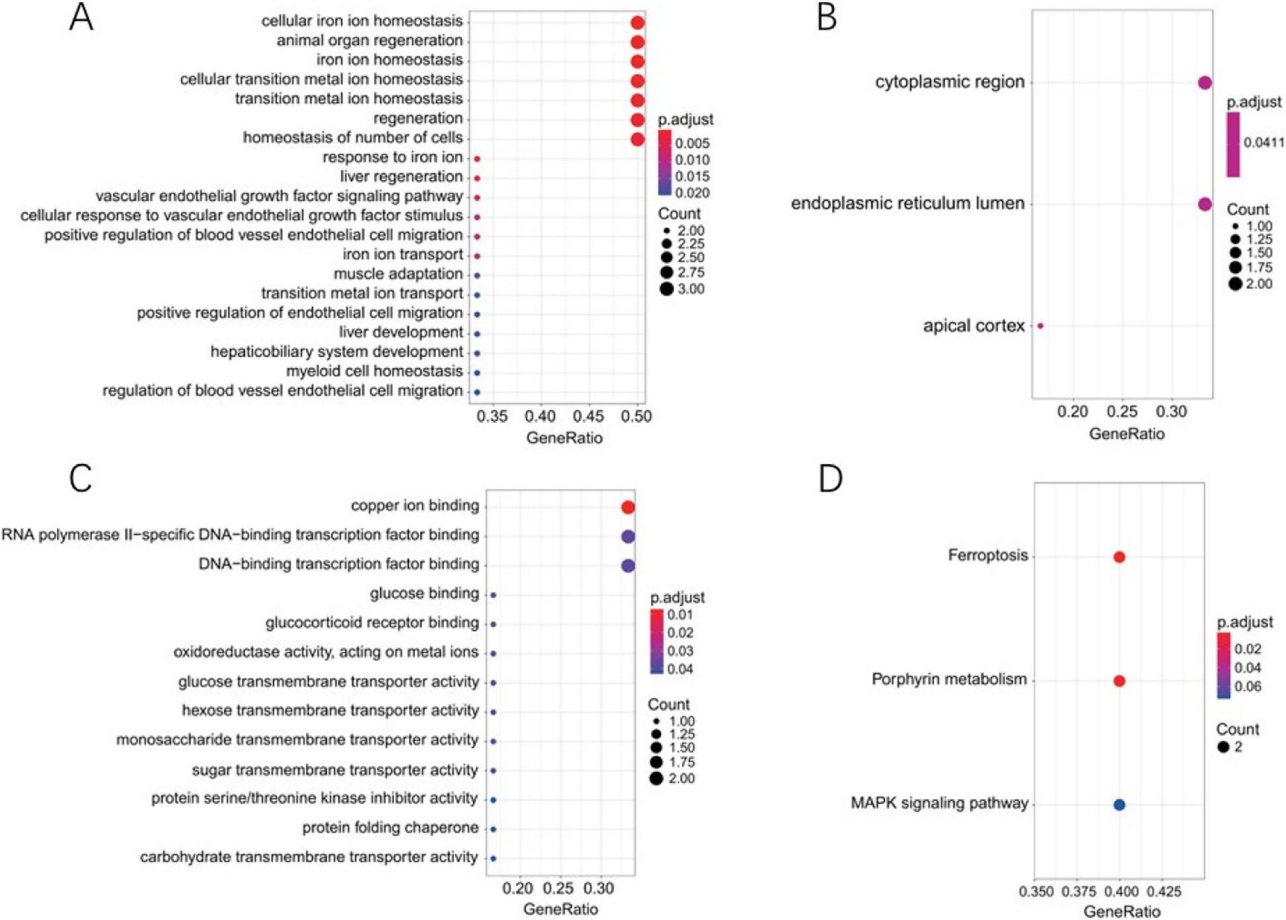
GO and KEGG enrichment analysis of ferroptosis-related DEGs. Note: **A** GO biological process enrichment results, **B** GO cell component enrichment results, **C** GO molecular function enrichment results, **D** KEGG pathway enrichment analysis bubble diagram. Bubble size indicates the number of genes associated with each term. The color of each bubble indicates the corrected *p*-value: the redder the color, the higher the enrichment

In order to understand the biological functions of osteoporotic ferroptosis-related DEGs more comprehensively, the GO and KEGG enrichment analysis of 6 ferroptosis-related DEGs were performed using the “clusterProfiler” package in R software. GO functional analysis shows they are mainly involved in related biological processes, such as cellular iron ion homeostasis, animal organ regeneration. They are components of related cells, such as cytoplasmic region, endoplasmic reticulum lumen, and simultaneously regulate related molecular functions such as copper ion binding, RNA polymerase II-specific DNA-binding transcription factor binding, the enrichment results were displayed using bubble plots respectively (Fig. 2A-C). KEGG pathway enrichment analysis showed that ferroptosis-related DEGs were mainly involved in Ferroptosis, Porphyrin metabolism, and MAPK signaling pathway-related signaling pathways (Fig. 2D).

### Random forest screening of key ferroptosis genes

**Fig. 3:**
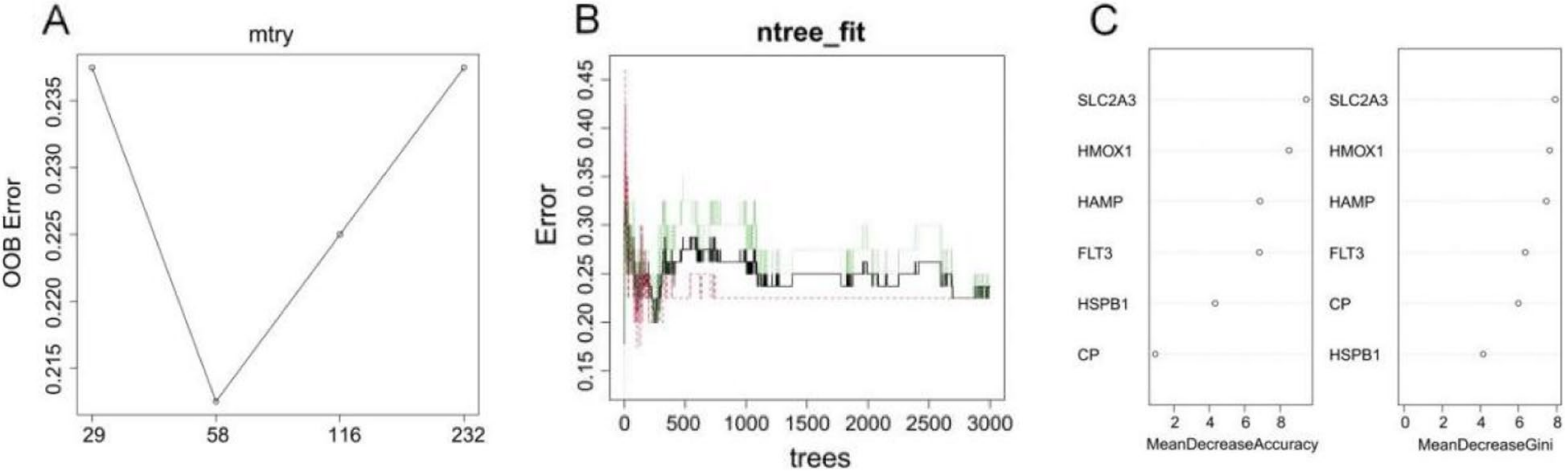
Random Forest Model. Note: **A** OOB error rate distribution at different mtry values. **B** OOB error rate distribution for different ntree values. **C** The six ferroptosis-related DEGs are sorted by the values of Accuracy and Gini, respectively

In order to further identify key ferroptosis genes, we performed random forest analysis on 6 ferroptosis-related differentially expressed genes. When the mtry value was 58 and ntree was 500, a random forest model was constructed to obtain the Mean Decrease Accuracy and Mean Decrease Gini values. Finally, five genes with Gini values greater than 5 were identified as key ferroptosis genes, including *SLC2A3*, *HMOX1*, *HAMP*, *FLT3* and *CP*. The results of the random forest model are shown in Fig. 3.

### Correlation analysis of key ferroptosis genes

**Fig. 4:**
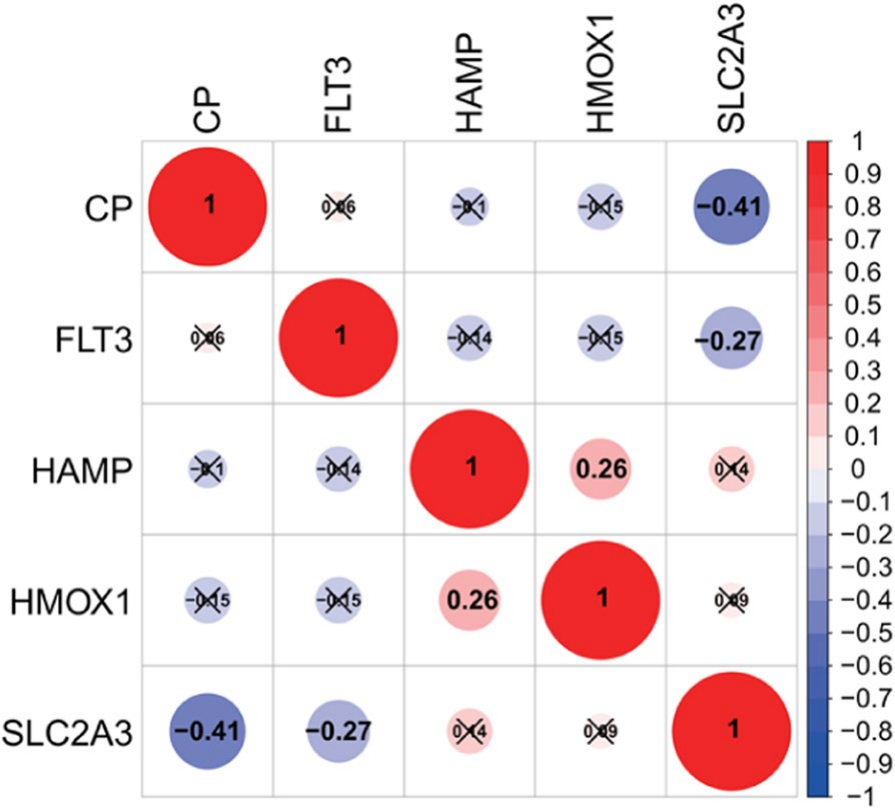
Expression correlations among key ferroptosis genes. Note: Red represents positive correlation, blue represents negative correlation; the size of the dots, the size of the value and the depth of the color represent the magnitude of the correlation; the cross mark represents the failure of the significance test

Correlation analysis was performed on the correlation heat map of the five key ferroptosis genes, and the results are shown in Fig. 4. In general, the closer the cor correlation coefficient is to 0, the smaller the correlation between genes, and the closer to 1, the more significant the correlation between genes. The results of gene correlation analysis showed that both *CP* (cor = -0.41) and *FLT3* (cor = -0.27) were significantly negatively correlated with *SLC2A3*, which indicated that the expression of *SLC2A3* and *CP* or *FLT3* genes showed mutual inhibition. In addition, there was a significant positive correlation between *HMOX1* and *HAMP* (cor = 0.26), which indicated that the expression of *HMOX1* and *HAMP* genes showed a mutual promotion effect. However, the cor correlation coefficients among the above genes are small, and the absolute values are all less than 0.5, indicating low correlation between genes.

### lncRNA-miRNA-mRNA-TF regulatory network

**Fig. 5:**
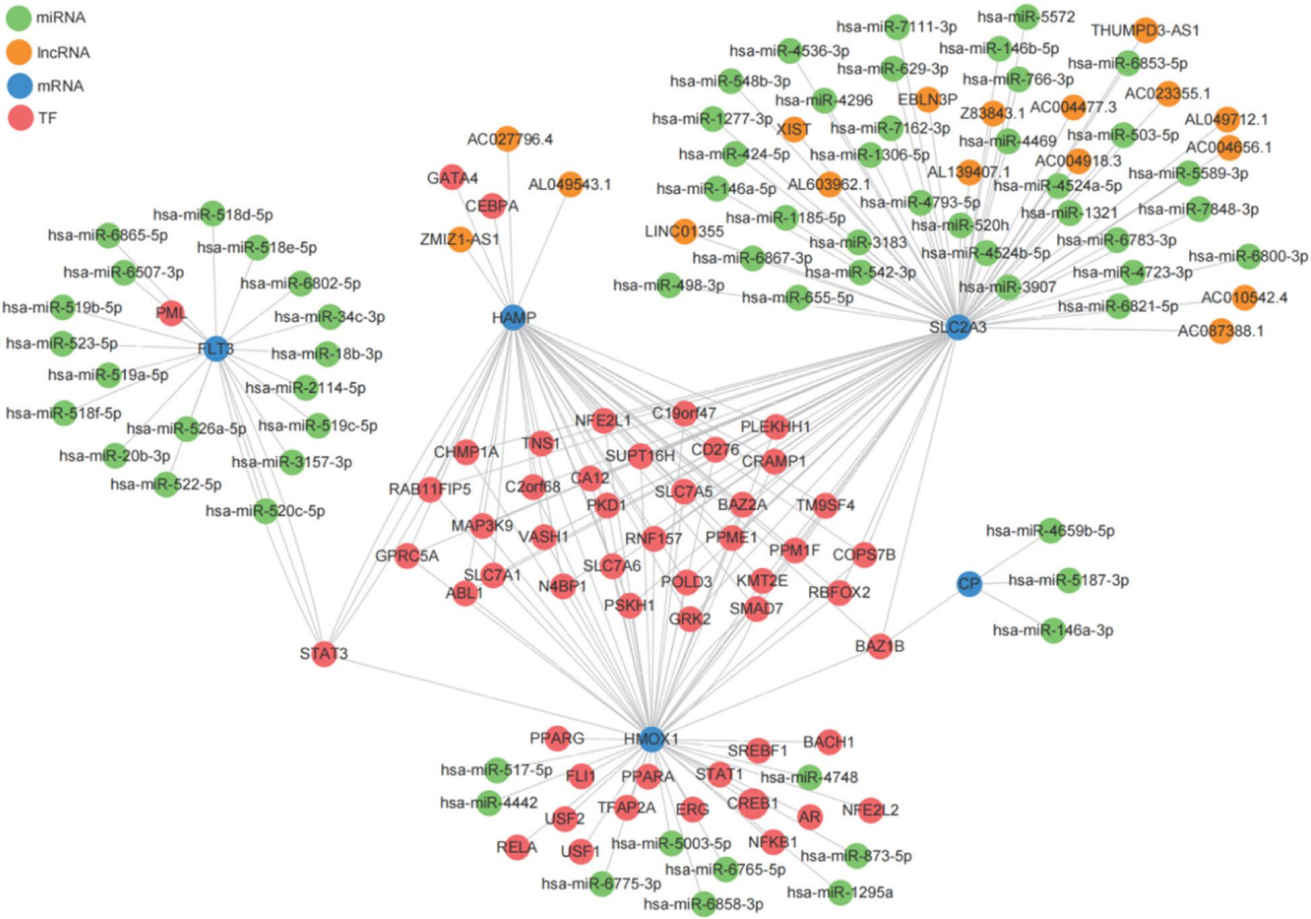
lncRNA-miRNA-mRNA-TF regulatory network of key ferroptosis genes. Note: Each node represents a gene and each line represents the gene interaction. Orange dots represent lncRNAs, red dots represent TFs, green dots represent miRNAs, and blue dots represent mRNAs. The network has 17 lncRNAs, 64 miRNAs, five mRNAs, and 52 TFs

The lncRNA-miRNA-mRNA-TF regulatory network was successfully constructed using Cytoscape software. The results are shown in Fig. 5. *STAT3* can interact with *FLT3*, *HAMP*, and *HMOX1* simultaneously, and *BAZ1B* can interact with *CP*, *SLC2A3*, *HAMP*, and *HMOX1* simultaneously. In addition, thirty-two TFs simultaneously interacted with *HAMP*, *SLC2A3*, and *HMOX1*, whereas both miRNAs and lncRNAs only interacted with a single key ferroptosis gene.

### ROC curve

**Fig. 6:**
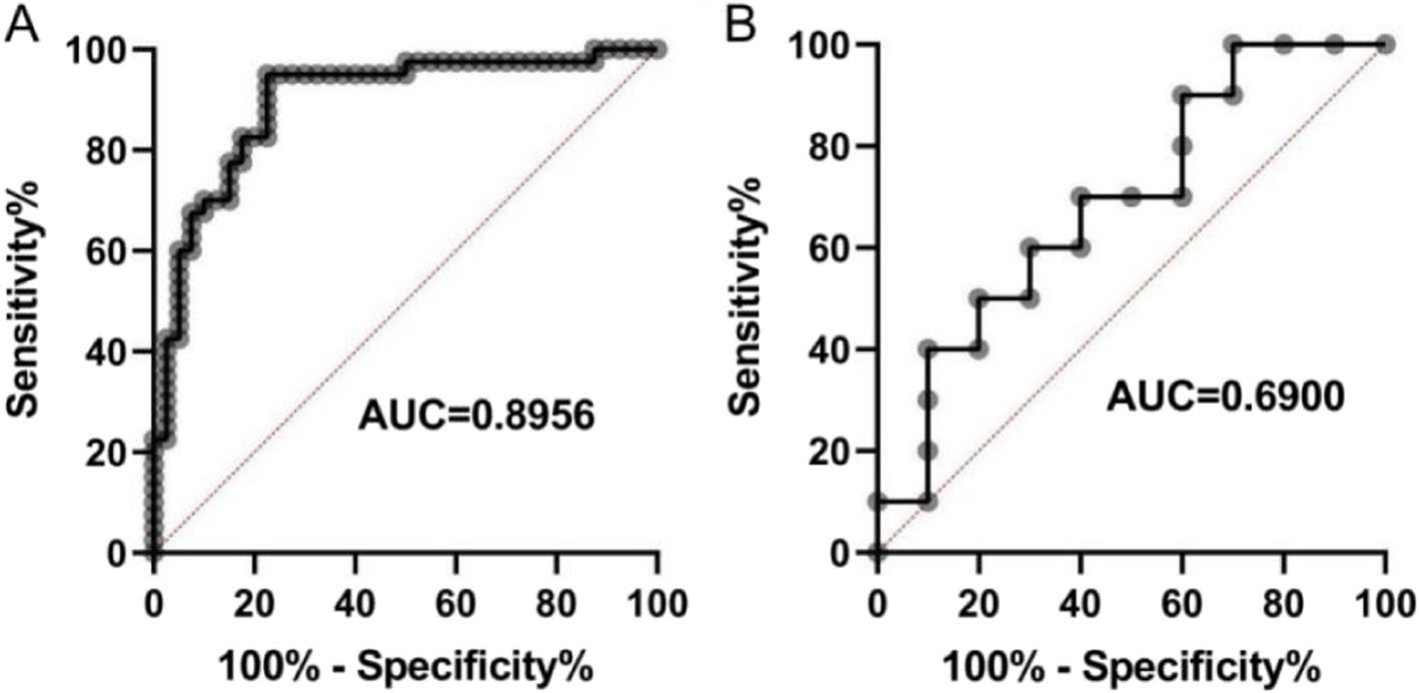
ROC curve. Note: **A** ROC curve of the diagnostic model in the GSE56815 training set. **B** ROC curve of diagnostic performance in GSE7429 validation set

Using the five key ferroptosis genes screened out by random forest, a disease diagnosis model was constructed, and the diagnosis model formula was obtained: Risk score = CP*5.40769938450489 + FLT3*2.24105334598503 + HAMP*(-1.67488503222183) + HMOX1*(-1.38903610456237) + *SLC2A* 3* (-1.78638247617311). After the model was constructed, ROC curve analysis was used to verify the validity and accuracy of the diagnostic model, and the AUC value of the diagnostic model was obtained as 0.8956 (Fig. 6A), indicating that the model had an excellent diagnostic performance. Finally, five key ferroptosis genes were used to build a disease diagnosis model in the external validation set GSE7429 to verify the diagnostic ability of the model. The results showed that the AUC value of the ROC curve was 0.6900 (Fig. 6B), which further indicated that the diagnostic effect of the model was perfect.

### Molecular docking screening for potential therapeutic small molecule compounds

**Fig. 7:**
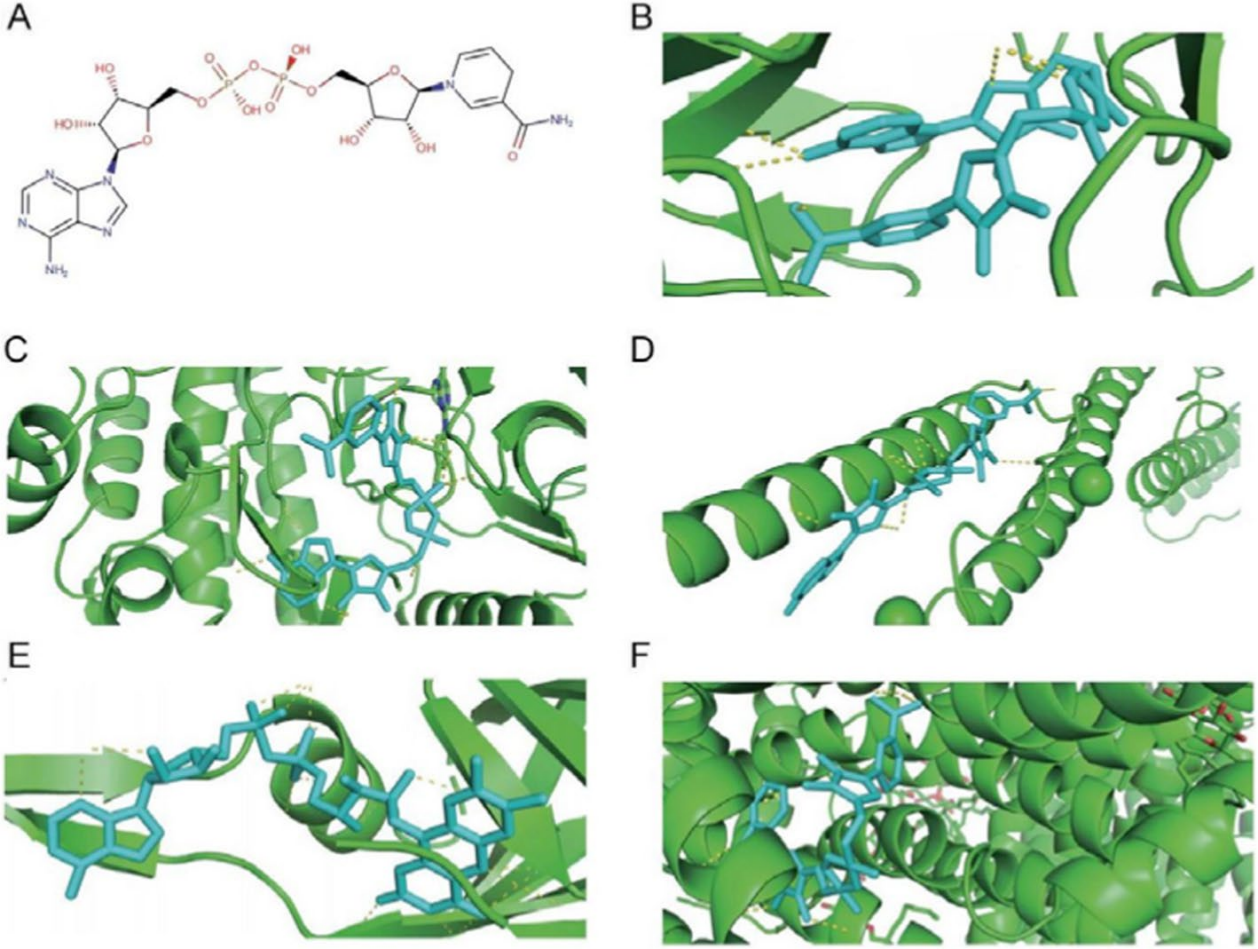
NADH molecular docking conformation. Note: **A** NADH compound structure. **B**-**F** are the docking conformations of NADH with *CP*, *FLT3*, *HAMP*, *HMOX1* and *SLC2A3* in sequence. Green represents large molecules (receptors), and cyan represents small molecules (ligands)

**Fig. 8:**
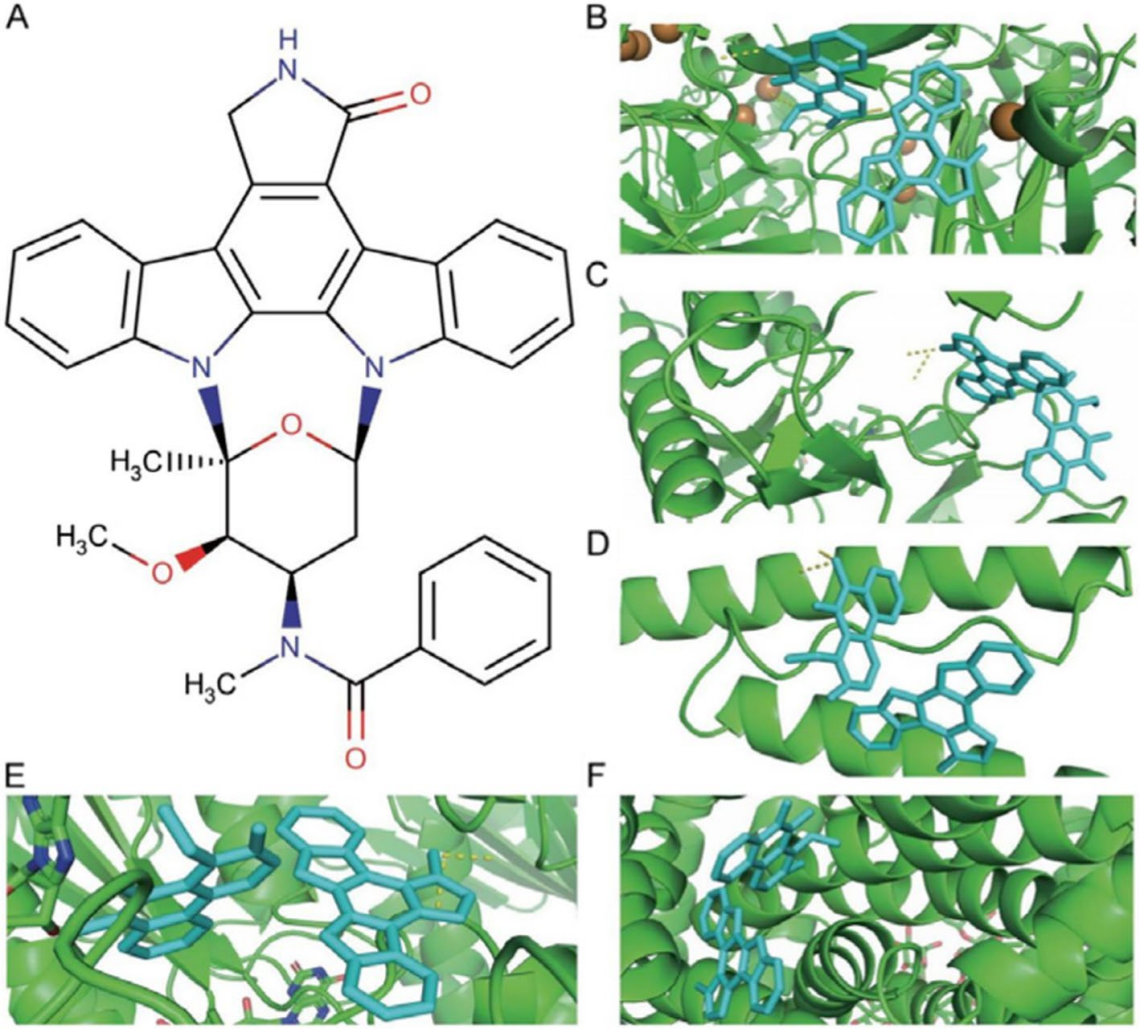
Midostaurin molecular docking conformation. Note: **A** NADH compound structure. **B**-**F** are the docking conformations of Midostaurin with *CP*, *FLT3*, *HAMP*, *HMOX1* and *SLC2A3* in sequence. Green represents large molecules (receptors), and cyan represents small molecules (ligands)

**Fig. 9:**
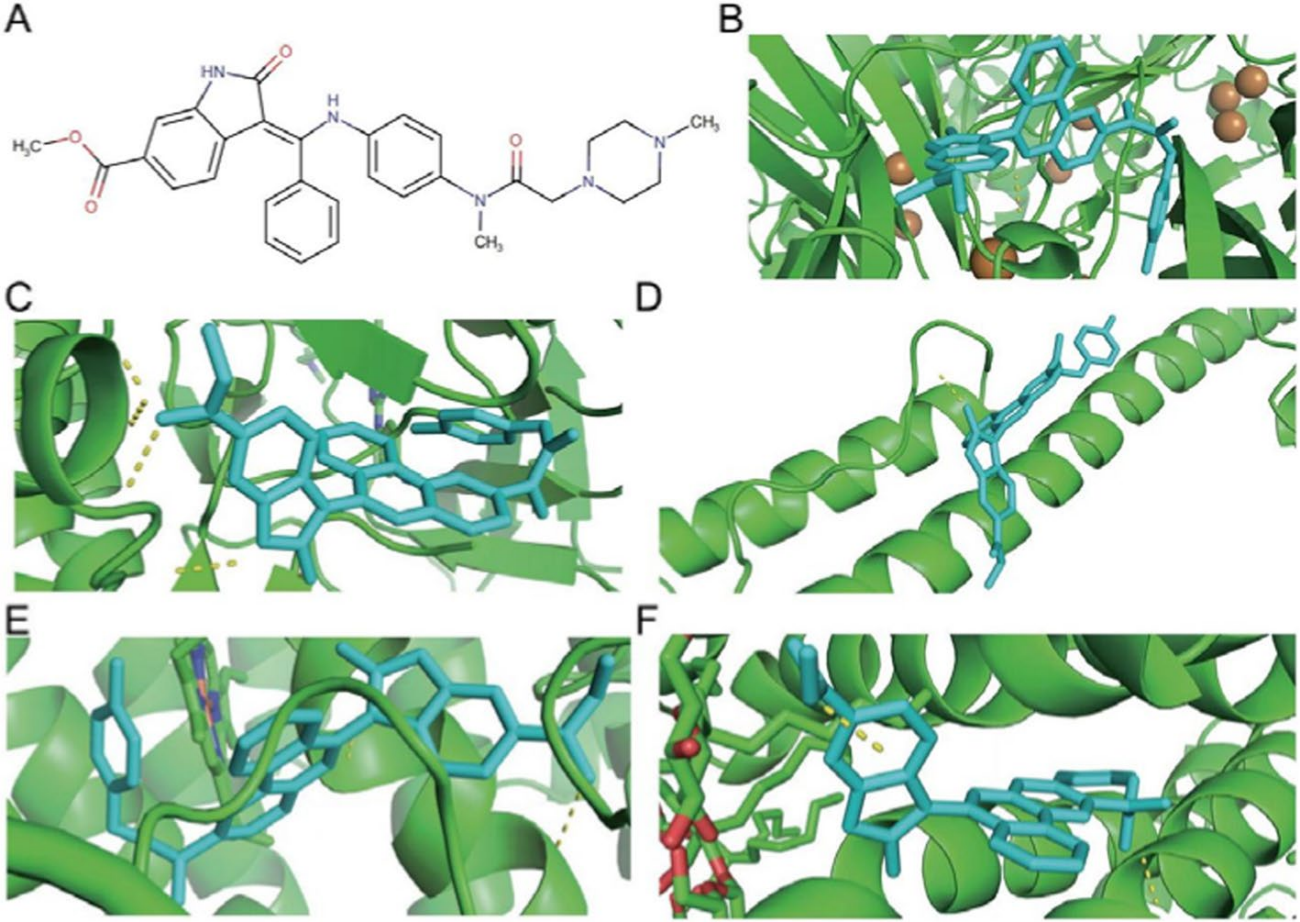
Nintedanib molecular docking conformation. Note: **A** NADH compound structure. **B**-**F** are the docking conformations of Nintedanib with *CP*, *FLT3*, *HAMP*, *HMOX1* and *SLC2A3* in sequence. Green represents large molecules (receptors), and cyan represents small molecules (ligands)

When screening small molecule compounds, select compounds that simultaneously perform molecular docking with five key ferroptosis genes and have a binding energy of Affinity ≤ -7 kcal/mol as candidates. Three small molecule compounds were obtained after the screening, including NADH (DrugBank ID: DB00157), Midostaurin (DrugBank ID: DB06595) and Nintedanib (DrugBank ID: DB09079). The results showed that the three candidate small molecule compounds all had good docking affinity to the five key ferroptosis genes (Table 2). The structural information and molecular docking conformation of small molecule compounds are shown in Figs. 7, 8 and 9, respectively.

**Table 2:** Molecular docking scores of NADH, Midostaurin, and Nintedani with five key ferroptosis genes (kcal/mol)

Compound/Gene	CP	FLT3	HAMP	HMOX1	SLC2A3
**NADH**	-9.7	-9.2	-7.2	-9.6	-9.3
**Midostaurin**	-11.1	-9.2	-7.8	-10.7	-11.9
**Nintedanib**	-8.9	-8.2	-7.2	-9.4	-11.1

### Micro-CT bone morphology detection

**Fig. 10:**
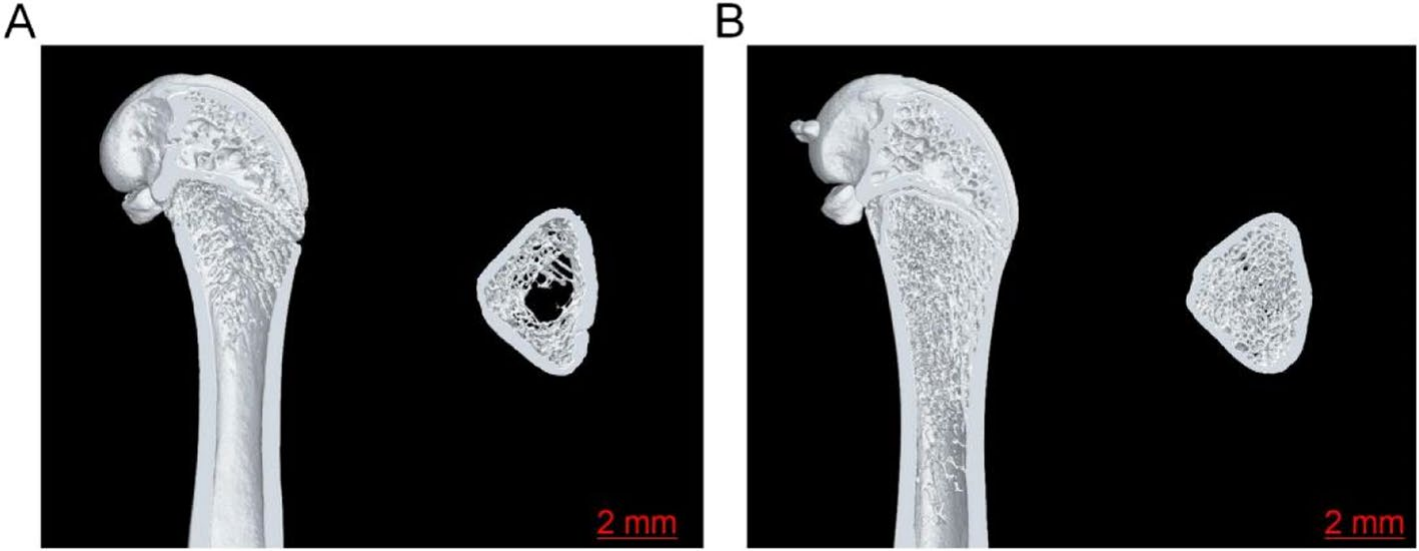
Micro-CT scan results. Figure **A** represents the Sham group, and Figure **B** represents the OVX group. Note: Micro-CT of rat proximal femur in coronal plane and cross-Sect. 5 mm below the growth plate

The results of the Micro-CT bone scan showed that compared with Sham, the OVX group had a thinner trabecular bone, significantly less trabecular bone, a large area of bone marrow without trabecular bone under the proximal femur, and an enlarged bone marrow cavity. Imaging shows the appearance of osteoporosis in Fig. 10.

### Micro-CT analysis of bone microstructural parameters

**Fig. 11:**
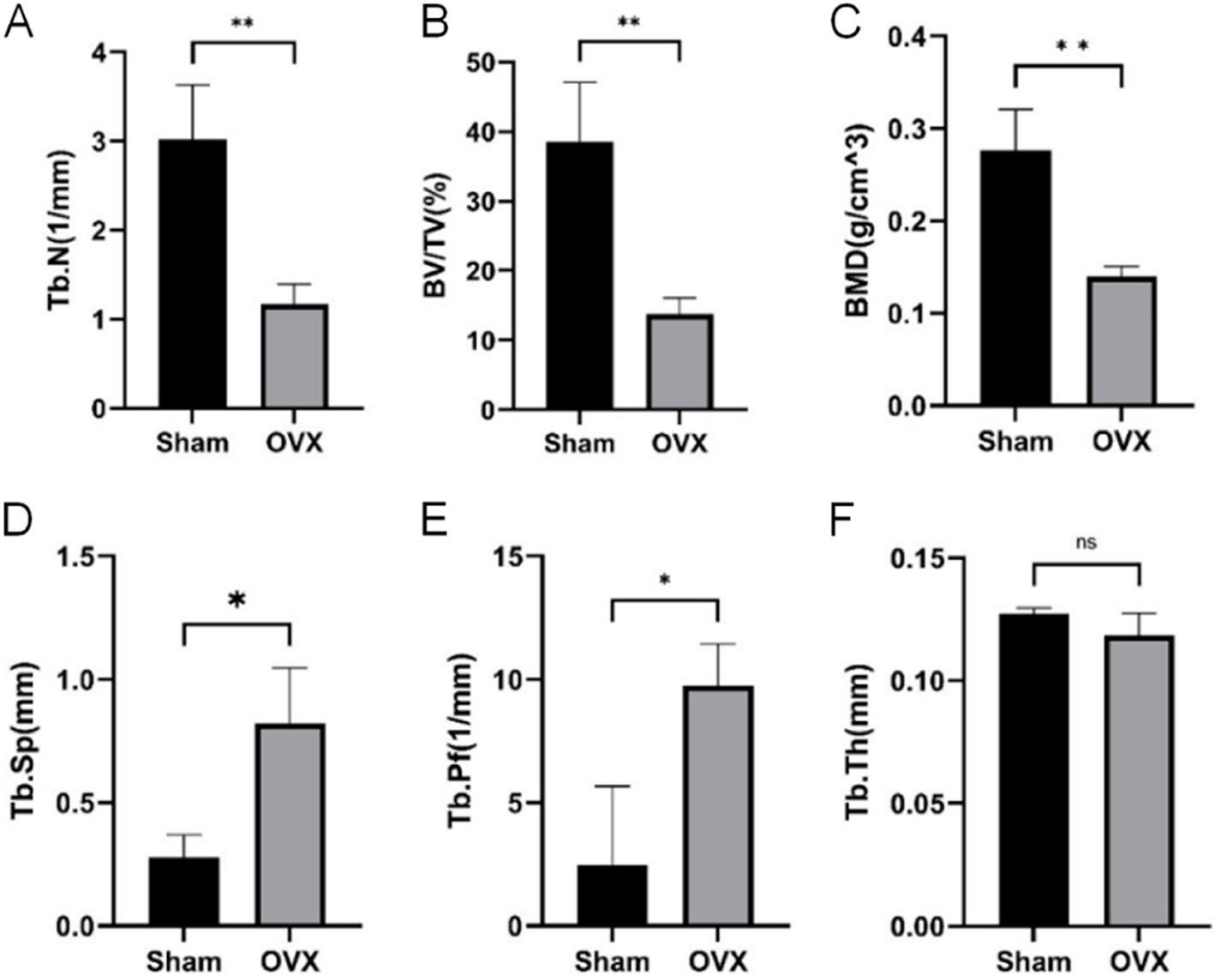
**A**-**F** shows the difference analysis of Micro-CT bone microstructural parameters (Tb.N, BV/TV, BMD, Tb.Sp, Tb.Pf, Tb.Th) between the Sham and OVX groups. *P < 0.05, **P < 0.01

Three-dimensional reconstruction was performed after Micro-CT scanning of the proximal femur in rats. It can be seen from the coronal layer-by-layer observation that compared with the Sham group (Fig. 11), the Tb.N, BV/TV, and BMD of the OVX group were significantly decreased (*P* < 0.01, *P* < 0.01, *P* < 0.01), while Tb.Sp, Tb.Pf increased significantly (*P* < 0.05, *P* < 0.05), and statistical analysis showed that the differences were statistically significant (Fig. 11A-E). However, Tb.Th in the OVX group decreased to a certain extent (*P* > 0.05), but the difference was not statistically significant (Fig. 11F). The results showed that the osteoporosis model in the OVX group was successfully established.

### qRT-PCR detection

**Fig. 12:**
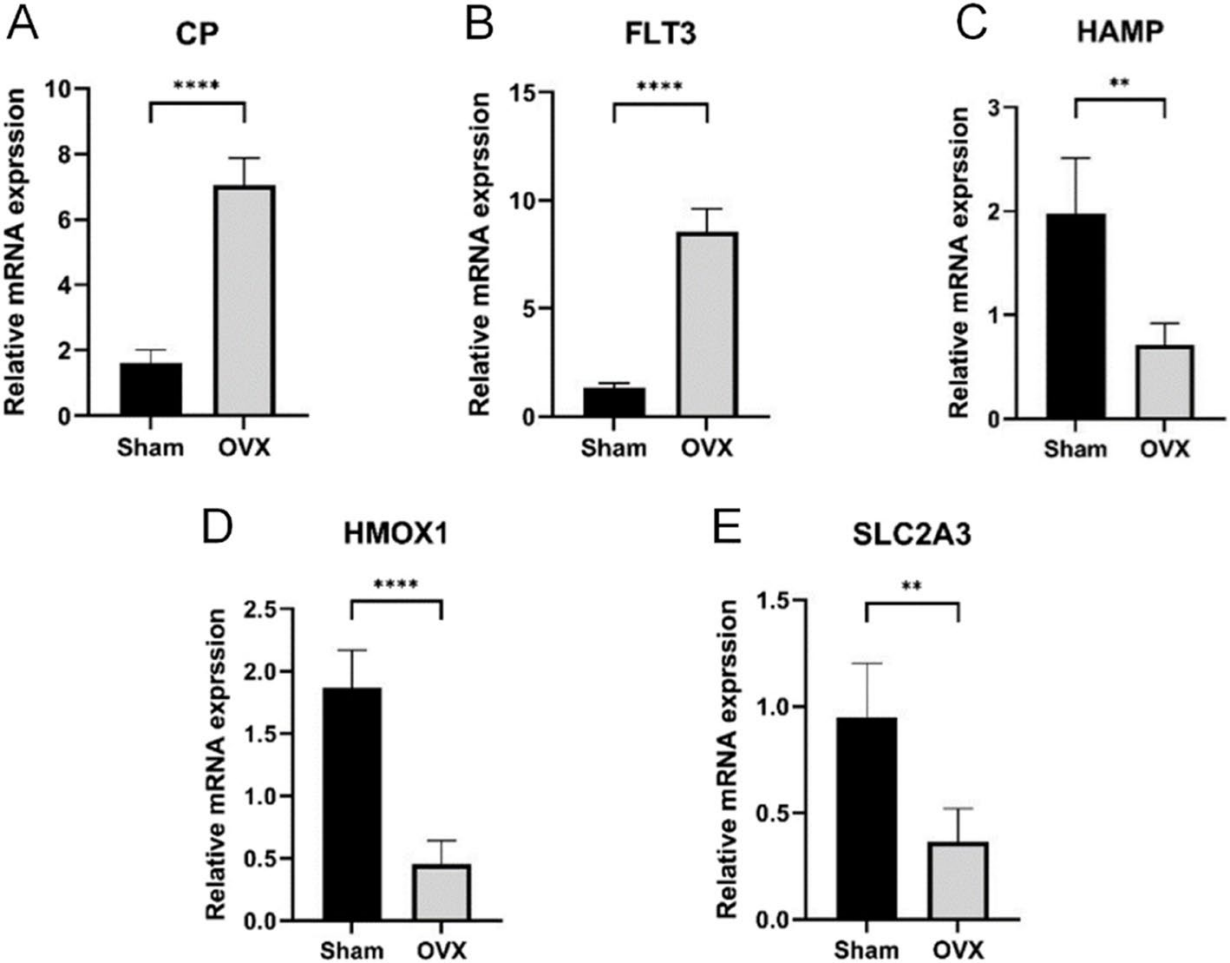
**A**-**E** show the differential analysis of the mRNA expression levels of five key ferroptosis genes (*CP*, *FLT3*, *HAMP*, *HMOX1*, and *SLC2A3*) in the Sham and OVX groups, respectively. **P < 0.01, ****P < 0.0001

In order to further verify the gene expression results of bioinformatics analysis, this study used qRT-PCR to detect the relative mRNA expression level of *CP*, *FLT3*, *HAMP*, *HMOX1*, and *SLC2A3* in the blood monocytes of the model group (OVX) and the sham operation group (Sham), and GAPDH was selected as an internal reference. Figure 12 shows that the mRNA expression levels of *CP* and *FLT3* in the OVX group were significantly increased compared with the Sham group. In addition, the mRNA expression levels of *HAMP*, *HMOX1*, and *SLC2A3* in the OVX group were significantly lower than those in the Sham group.

## Discussion

At present, the pathogenesis of osteoporosis is still unclear. Many studies believe that osteoporosis is closely related to various factors such as loss of bone homeostasis, autophagy, abnormal iron metabolism, aging, and gut microbiota [27]. In recent years, the emergence of ferroptosis has provided new clues for further understanding the pathogenesis of osteoporosis [28].

This study mainly used bioinformatics technology to screen ferroptosis-related biomarkers on the osteoporosis chip data set. Six DEGs related to ferroptosis (including *CP*, *HAMP*, *HMOX1*, *FLT3*, *SLC2A3*, and *HSPB1*) were preliminarily screened out by differential analysis on the GSE56815 dataset containing 80 samples in the GEO database. GO functional enrichment analysis showed that ferroptosis-related DEGs were mainly enriched in cellular iron homeostasis, cytoplasmic region, copper ion binding, etc. In recent years, studies have reported that iron homeostasis is of great significance in osteoporosis, especially in postmenopausal women, iron overload is closely related to osteoporosis [29]. In addition, studies have reported that iron homeostasis and iron regulation play an essential role in ferroptosis [30]. KEGG pathway enrichment analysis results showed that ferroptosis-related DEGs were mainly related to ferroptosis and porphyrin metabolism pathways. Studies have reported that activation of signal transducer and activator of transcription 3 (STAT3) can reduce ferroptosis and promote osteoclast differentiation, which indicates that ferroptosis is important in osteoporosis by regulating osteoclast differentiation [31]. By analyzing the function and pathway of ferroptosis-related DEGs, we found that iron ions played an irreplaceable role in it, and iron ions are closely related to ferroptosis. Therefore, the relationship between ferroptosis and osteoporosis deserves further research, which will help further understand osteoporosis’s pathogenesis.

In this study, the random forest model was used to screen out five key ferroptosis genes, including *CP*, *HAMP*, *HMOX1*, *FLT3*, and *SLC2A3*, and these 5 differentially expressed key ferroptosis genes were preliminarily verified in the osteoporosis model constructed. A diagnostic model was built by LASSO regression, which demonstrated that the five key ferroptosis genes performed well in diagnosing osteoporosis. Correlation analysis of key ferroptosis genes showed that *HMOX1* was significantly positively correlated with *HAMP* co-expression, and *CP* and *FLT3* were significantly negatively correlated with *SLC2A3* co-expression. Ceruloplasmin (*CP*), this gene can encode a metalloprotein, which can not only bind most copper in plasma, but also participate in the process of oxidation of ferrous iron to ferric iron [32]. As an antioxidant, it may lead to a variety of diseases [33, 34], it has been found that the loss of ceruloplasmin can induce systemic iron deficiency and disrupt iron homeostasis [35]. Loss of *CP* promotes erastin- and RSL3-induced ferroptosis and causes excessive accumulation of intracellular ferrous ions (Fe^2+^) and lipid reactive oxygen species (ROS) [36]. In addition, studies have found that reducing iron overload can improve bone cell metabolism and growth in vitro and in vivo. Given the significant iron overload found in aging populations, especially in women, reducing iron overload has some potential in treating osteoporosis [29]. Studies have found that excessive iron accumulation is a risk factor for osteopenia and osteoporosis. Osteoporotic bone loss caused by excessive iron accumulation is driven by osteoblast ferroptosis triggered by NOX4 (NADPH oxidase 4) [37]. Hepcidin Antimicrobial Peptide (*HAMP*) is an iron homeostasis regulator. It has been reported in the literature that hepcidin deficiency is directly related to bone loss, which may induce osteoporosis [38]. In addition, studies have found that high hepcidin expression induces a decrease in iron content and negatively regulates osteoclast differentiation, which plays a protective role in the pathogenesis of postmenopausal osteoporosis [39]. Heme Oxygenase 1 (*HMOX1*) is an essential enzyme that catalyzes heme degradation. It not only has anti-inflammatory and anti-oxidative stress effects, but also plays an anti-apoptotic role. *HMOX1*-activated *HO-1* is associated with the prevention of various diseases, including cancer, diabetes, cardiovascular disease, and osteoarthritis [40–43], and current studies have shown that *HMOX1* may play a role in maintaining bone homeostasis, increased expression of *HMOX1* can increase the levels of osteopontin (OPN), osteoprotegerin (OPG) and bone morphogenetic protein-2 (BMP-2) [44]. Inhibition of *HMOX1* activity can prevent the increase of osteonectin and accelerate the decrease of osteocalcin and OPG in a high-glucose environment. High glucose can cause bone marrow mesenchymal stem cells (MSC) to weaken bone differentiation, while targeting *HMOX1* gene expression weakens this process [45]. This study shows that *HMOX1* is low-expressed in osteoporosis, and this gene may promote osteoporosis development by reducing MSC’s osteogenic differentiation ability and regulating bone metabolism. *FLT3*, also known as Fms Related Receptor Tyrosine Kinase 3, is a member of class III receptor tyrosine kinases, expressed on the cell surface of hematopoietic progenitor cells, and involved in regulating the maintenance, Proliferation, and differentiation. It has been reported that *FLT3* is associated with low bone mineral density and fracture risk in postmenopausal women, but the specific mechanism is still unclear [46]. *SLC2A3* is also known as Solute Carrier Family 2 Member 3, its main function is to promote the activity of glucose transporter, and it can also mediate the uptake of various other monosaccharides on the cell membrane [47]. However, there are few literature reports about its relationship with osteoporosis, so its mechanism of action in osteoporosis needs further research and exploration.

In this study, we constructed a lncRNA-miRNA-mRNA-TF regulatory network. Through these five key ferroptosis genes, we found possible interacting molecules. The results showed that *BAZ1B* can interact with *CP*, *SLC2A3*, *HAMP*, and *HMOX1* at the same time, while *STAT3* can interact with *FLT3*, *HAMP*, and *HMOX1* simultaneously. It has been reported in the literature that *STAT3* and its network are involved in bone remodeling and the development of osteoporosis [48], and some studies have found that *STAT3* can play an essential role in maintaining bone development and homeostasis by regulating osteoblasts [49]. Given the effect of *STAT3* on bone homeostasis, we believe that *BAZ1B* may also play an important role in osteoporosis. At present, there are few literature reports on the role of *BAZ1B* in osteoporosis. Therefore, the relationship between *BAZ1B* and osteoporosis needs to be further explored. Currently, there are few studies on the relationship between *STAT3* and *BAZ1B* and interacting genes. Further research is needed in the future.

We successfully excavated three small molecule compounds (NADH, Midostaurin, and Nintedanib) from the DurgBank database through molecular docking technology. Midostaurin is a multi-target protein kinase inhibitor, and its targets include *FLT3*, KIT, PDGFRA, PDGFRB, and VEGF receptors (KDR and FES). Studies have shown that the drug can treat leukemia and *FLT3*-mutated mastocytosis [50, 51]. Nintedanib belongs to a class of tyrosine kinase inhibitors that treat interstitial lung disease associated with systemic sclerosis [52]. Side effects such as diarrhea, nausea, abdominal pain, vomiting, elevated liver enzymes, loss of appetite, headache, and high blood pressure may occur when using Nintedanib. NADH is a strong antioxidant in cells, which can protect cells by inhibiting lipid peroxidation. NADH is the reduced form of NAD^+^, and NAD^+^ is the oxidized form of NADH. Current studies have shown that oral NAD supplementation improves memory and attention in some healthy individuals, but its efficacy has yet to be proven [53]. However, there are currently no literature reports on the relationship between the three small molecular compounds and osteoporosis.

In general, the innovation of this study lies in the use of bioinformatics analysis and molecular docking technology to screen ferroptosis-related genes and small molecular compounds that interact with them. At present, there are few studies on osteoporosis and ferroptosis, especially the screening of potential drugs for osteoporosis-related to ferroptosis by molecular docking technology. However, this study also has certain limitations. Postmenopausal osteoporosis is a good preclinical model among osteoporosis rat models. The ovariectomized osteoporosis rat model simulates bone loss caused by estrogen deficiency and exhibits clinical manifestations of postmenopausal osteoporosis. In this study, the verification of the expression levels of key ferroptosis genes was conducted in an ovariectomized osteoporosis rat animal model, and further experimental verification is needed in the human population. The verification of the expression level of key ferroptosis genes was carried out in animal models, and further experimental verification in the population is required.

## Conclusion

In this study, five key ferroptosis genes (*CP*, *FLT3*, *HAMP*, *HMOX1*, and *SLC2A3*) were identified as biomarkers related to OP ferroptosis, and three small molecular compounds (NADH, Midostaurin, Nintedanib) were screened out may be potential therapeutic compounds related to OP ferroptosis, which provides us with new insights into the pathogenesis and treatment of OP.

### Supplementary Information

**Supplementary Material 1.**

**Supplementary Material 2.**

## Data Availability

The datasets used and/or analysed during the current study are available from the corresponding author on reasonable request. In addition, you can also get datasets from the websites (https://starbase.sysu.edu.cn/) (https://www.grnpedia.org/trrust/) (https://go.drugbank.com/) (http://ophid.utoronto.ca/mirDIP/) (http://www.zhounan.org/ferrdb/index.html).
